# Cases of Wounded and Diseased Arteries

**Published:** 1827-03

**Authors:** B. Travers

**Affiliations:** St. Thomas's Hospital


### WOUNDED ARTERIES.

*Cases of Wounded and Diseased Arteries, treated principally at* St. Thomas's Hospital, *by* B. Travers, Esq. F.R.S.

The following are short notes of cases of wounded or diseased arteries. I shall not detail such as are either of ordinary occurrence, or present no peculiar circumstance in their history, symptoms, or treatment. Of the latter a considerable proportion has fallen to my share; but the case of femoral or popliteal aneurism is so familiar, and the operation which constitutes its cure so well established and successful, that the repetition of the narrative would be an useless tax upon the reader's patience.

### Case I. *of Tumor, supposed to be Aneurism, spontaneously cured.*

May 17th, 1817.— —— Hallett, æt. thirty, a tailor, of healthy appearance. About three weeks prior to this date, the day after a very long walk, he perceived a swelling in the right ham, which has gradually increased in size, and at first had a distinct, and even strong pulsation. The tumor is defined, but nearly fills the popliteal space, and has the character of aneurism, excepting that the pulsation is obscure. There is stiffness and pain in the part, and he cannot extend the leg. The joint is in no degree affected, nor has he a swelling in any other part of his body.

June 1st.—Since his admission and confinement in a horizontal posture, the tumor has contracted in its dimensions, and become firmer; not the slightest pulsation is perceptible, and the almost complete extension of the limb can be performed. No application whatever has been made since his admission. The man describes the change to be such as confirms the belief that the tumor was aneurismal.

A soap plaster was applied to the ham, and the whole extremity bound with rollers from the instep to the hip.

Within a month the man left the hospital free from lameness, and with scarce a vestige of the tumor. He promised to return in the event of any alteration, but did not again present himself.

This might, or might not, be aneurism. It presented the external character of that disease, and certainly not that of bursal, glandular, or cellular tumor. The pulsation at the time of the man's admission was not strong enough to be pronounced, direct. He stated that it was much weaker than it had been. The contraction of the swelling was uniform, or, on its whole circumference, not broken; and the tension was exchanged for a greater density. It was believed by those who examined it to be aneurism. If not, it may fairly be asked, what was it?

CASE II. *Blood Tumor, from an ulcerated Opening of the Femoral Artery, which proved fatal.*

A stout man, of middle age, was admitted under the late Mr. CHANDLER, into St. Thomas's Hospital, with a diffused deep-seated swelling of the thigh, of great size and tension, but without pulsation or any original character of aneurism. It was reported to have increased rapidly of late. Shortly after his admission, while the history and treatment of the case were under deliberation, he died suddenly in the night.

The dissection of the limb showed that an opening in the femoral artery had permitted the escape of blood, by repeated issues, to such extent as to occasion the enormous increase of the swelling, and ultimately the fatal syncope. Lamellated coagula formed the walls of the tumor, but there was no vestige of a sac.

Since this case occurred, I have felt jealous of swellings which, void of pulsation, have had the character of spurious aneurism, from the depth, diffusedness, and tension of the swelling; which are, in fact, such as to disfigure the whole limb from joint to joint. The following case is in point.

CASE III. *of Blood Tumor, from an Ulcerated Opening in the Popliteal Artery, diffused over the greater part of the Thigh; for which Amputation was performed immediately below the Trochanter Minor.*

Richard Durrant, a farmer's labourer, æt. forty-five, was admitted into St. Thomas's Hospital, on September 1st, 1825, with a tumor occupying the lower three-fifths of the right thigh, most considerable on the inner side, rising on each side of the femur, leaving a central depression, as if confined by the attachments of the fascia to the ridges running from the linea aspera to the condyles. The integuments covering the tumor are very much discoloured and desquamating; and at the upper and inner part are two small openings, from which a sanious fluid is discharged. The foot and leg are much swollen and œdematous, and the cuticle peels off as if the limb had suffered from an attack of erysipelas. The man's countenance is pallid and haggard, and his lips are perfectly bloodless.

To the inquiries made concerning the origin of the disease, he gave very confused and incoherent replies, from which the following account was collected:—About nine weeks since, he was engaged in mowing grass, and, his shoes being unsound, he took cold; his feet inflamed, and a number of red spots appeared on his legs. A few days after this, while sitting before the fire, he felt something give way and trickle down his right thigh, and, but for immediate support, would have fallen to the ground. The swelling then began: he is certain it took place from below upwards, but is not quite clear whether the leg or thigh was first swollen. When asked as to pulsation, he at first said there was none; but afterwards corrected himself. There is at this time no fluctuation in the tumor; but a sufficient degree of elasticity to throw considerable doubt on the nature of the case; some supposing it to be an abscess following erysipelas, others diffused aneurism of the femoral artery. Mr. Travers gave his opinion that it was either diffused aneurism, or a tumor of the malignant fungoid species.

Sept. 2d.—℞. Ol. Ricini ℥ ss. statim sumend.

A probe introduced at the openings passed freely in every direction, and, when withdrawn, was followed by a sanious discharge unmixed with pus. In the evening, at least a pint of dark-coloured blood escaped from the openings; but further hemorrhage was readily suppressed. This occurrence led to the determination of operating early.

3d.—Doubts being still entertained as to the nature of the tumor, it was resolved to puncture it, and, if blood only escaped from the aperture, to proceed to amputate immediately. A scalpel was therefore plunged into the centre of the swelling, and, the finger being introduced at the opening, a quantity of grumous blood was discharged. The limb was then amputated, Mr. Key commanding the artery at the groin. Flaps were formed from the outer and inner sides of the thigh, and the bone sawn through immediately below the trochanter minor. The femoral, profunda, and superior perforating arteries required ligatures. Not more than one or two ounces of arterial blood were lost during the operation. The flaps, when brought together, formed a tolerably even line of union, about nine inches long.

On examination, the tumor was found to consist of coagulated blood diffused among the muscles of the thigh, which were much discoloured and partially disorganised. In one part of the tumor suppuration had commenced. In the centre of the popliteal space was an opening in the artery, half an inch long, of an elliptical oval shape, the edges being rounded off by ulceration. No traces of an aneurismal sac could be found.

Sept. 4th.—Passed a bad night. Extreme restlessness, and spasm of the stump; fever; furred tongue; quick, small pulse; frequent vomiting; cough rather troublesome.

℞. Haust. Salin. Effervesc. c. Tr. Opii m. v. quartis horis sumend.—Sago and Syrup.

The above unpleasant symptoms soon subsided, leaving him in a state of great debility.

From his wife, who visited the hospital to-day, we learned that, while sitting in his chair, on the first attack of the disease, he cried out, became very pale, and fell down fainting. The thigh suddenly swelled enormously, so as render it necessary to cut off his clothes. She described the pulsation by raising and depressing her hand quickly. After two or three days, the pulsation ceased as the swelling increased.

7th.—The wound partially dressed: the upper third has united by adhesion; healthy suppuration in moderate quantity.

9th.—℞. Dec. Cinch. c. Tr. ter die.—Vin. Rub. ℥ iij. indies.—Mutton-broth. A sinus is forming at the inner side of the stump.

14th.—The two lower ligatures came away. The stump is healthy, and healing rapidly; health improving; has a tolerable appetite; bowels regular.

Mutton-chop three times a-week; Porter ℔j. daily.

18th.—The last ligature came away. Wound healing rapidly. By the aid of compresses, the sinus is nearly obliterated.

28th.—Wound rather indolent, though much progress has been made; general health not quite so good.

℞. Quininæ Sulph. gr. ij. ex Infusi Rosæ ℥ j. ter die.—Applicetur vulneri Lotio Zinci. Sulphat.

October 3d.—Wound healed, except at the space left for the escape of pus from the sinus: here cicatrisation is rapidly proceeding.

15th.—Wooden leg. A few days afterwards he left the hospital quite well, and able to use his wooden leg with tolerable advantage.

The above was a case of much interest. In the case of bursten artery or aneurismal sac, which becomes in reality a blood tumor, amputation is commonly the only resource. Gangrene of the entire limb would, I imagine, be the inevitable result of applying a ligature upon the main artery of a limb laden with such a mass of unorganisable coagulum and disorganised structure. But, if this course were resolved upon, the best practice would be to lay open the tumor by incision, clear out the coagula, and tie the artery above and below the rupture, as in the old operation for aneurism.

(To be continued.)

